# 数字PCR检测慢性髓细胞性白血病患者国际标准化BCR::ABL1（P210）mRNA的室间比对

**DOI:** 10.3760/cma.j.cn121090-20260123-00054

**Published:** 2026-07

**Authors:** 亚溱 秦, 道新 马, 瑶 姚, 勇梅 朱, 阳丽 张, 虹 刘, 永杰 唐, 素霞 耿, 纯 乔, 成雷 王, 美兰 张, 继红 张, 利 姚, 小青 李, 剑锋 周, 倩 江

**Affiliations:** 1 北京大学人民医院、北京大学血液病研究所，北京 100044 Peking University People's Hospital, Peking University Institute of Hematology, Beijing 100044, China; 2 山东大学齐鲁医院血液科，济南 250012 Department of Hematology, Qilu Hospital of Shandong University, Jinan 250012, China; 3 中国医学科学院血液病医院（中国医学科学院血液学研究所）血液病理诊断中心，天津 300020 State Key Laboratory of Experimental Hematology, Institute of Hematology & Blood Diseases Hospital, Chinese Academy of Medical Sciences & Peking Union Medical College, Tianjin 300020, China; 4 上海交通大学医学院附属瑞金医院、上海血液学研究所，上海 200025 Shanghai Institute of Hematology, Ruijin Hospital Affiliated to Shanghai Jiao Tong University School of Medicine, Shanghai 200025, China; 5 重庆医科大学附属第一医院临床分子医学检测中心，重庆 410006 The Center for Clinical Molecular Medical Detection, Laboratory Medicine Center, The First Affiliated Hospital of Chongqing Medical University, Chongqing 400016, China; 6 新疆维吾尔自治区人民医院血液病科，乌鲁木齐 830001 Department of Hematology, People's Hospital of Xinjiang Uygur Autonomous Region, Urumqi 830001, China; 7 陆军军医大学第二附属医院血液病医学中心，重庆 400037 Medical Center of Hematology, the Second Affiliated Hospital of Army Medical University, Chongqing 400037, China; 8 南方医科大学附属广东省人民医院血液内科，广州 510080 Department of Hematology, Guangdong Provincial People's Hospital, Southern Medical University, Guangzhou 510080, China; 9 南京医科大学第一附属医院、江苏省人民医院血液科，南京 210029 Department of Hematology, the First Affiliated Hospital of Nanjing Medical University, Jiangsu Province Hospital, Nanjing 210029, China; 10 北京大学人民医院青岛医院血液中心，青岛 266100 Peking University People's Hospital Qingdao Hospital, Qingdao 266100, China; 11 华中科技大学同济医学院附属同济医院血液内科，武汉 430030 Department of Hematology, Tongji Hospital, Tongji Medical College, Huazhong University of Science and Technology, Wuhan 430030, China; 12 中国医科大学附属盛京医院血液研究室，沈阳 110022 Hematology Laboratory, Shengjing Hospital of China Medical University, Shenyang 110022, China; 13 苏州大学附属第一医院血液病研究室，苏州 215031 Hematology Research Laboratory, the First Affiliated Hospital of Soochow University, Suzhou 215031, China; 14 华中科技大学同济医学院附属协和医院血液科干细胞室，武汉 430022 Department of Hematology, Union Hospital, Tongji Medical College, Huazhong University of Science and Technology, Wuhan 430022, China; 15 天津金域医学检验实验室，天津 300392 Tianjin Kingmed Diagnostics Laboratory Co.Ltd, Tianjin 300392, China

**Keywords:** 白血病，髓细胞性，慢性, 室间比对, 数字PCR, 国际标准化BCR::ABL1 mRNA水平, 转换系数, Leukemia, myeloid, chronic, Inter-laboratory comparison, Digital PCR, BCR::ABL1^IS^, Conversion factor

## Abstract

**目的:**

通过室间样本派发比对评估数字PCR（dPCR）检测慢性髓细胞性白血病患者国际标准化BCR::ABL1（P210）mRNA（BCR::ABL1^IS^）的能力。

**方法:**

本研究为多中心比对实验研究。采用待废弃的临床样本统一制备共37种不同BCR::ABL1^IS^水平的比对样本，其中22种用于实时定量PCR（RQ-PCR）检测以进行转换系数（CF）的再验证，其余15种加上用于RQ-PCR的6种用于dPCR比对。北京大学人民医院通过检测WHO国际标准品判定自身dPCR及RQ-PCR的CF的有效性。通过Bland-Altman一致性分析判断各实验室CF的有效性，若无合格CF，则重新计算并评估有效性。

**结果:**

参加dPCR比对的14家实验室中13家所有比对样本的BCR::ABL1均能检出。参加RQ-PCR比对的13家实验室中5家CF合格，其余8家重新计算的CF均合格。dPCR比对中，11家采用同一商品化试剂盒，10家合格；1家采用自配试剂并利用国家临床检验中心标准品获得CF亦合格；无CF的2家重新计算的CF均合格。同时进行dPCR和RQ-PCR比对的样本结果比较显示，各家采用各自合格CF得出的BCR::ABL1^IS^结果差异均无统计学意义（均*P*≥0.1）；但RQ-PCR原有CF不合格的6家实验室中，2家的dPCR检测结果与由原CF得出的BCR::ABL1^IS^结果之间差异有统计学意义（均*P*<0.05），3家趋于差异有统计学意义（0.05<*P*<0.1）。

**结论:**

dPCR检测BCR::ABL1^IS^具有高敏感性、高准确性以及与RQ-PCR结果的高度一致性。

## Abstract (en)

**Objective:**

To evaluate the capability of digital PCR（dPCR）in detecting the internationally standardized BCR::ABL1（P210）mRNA（BCR::ABL1^IS^）of patients with chronic myeloid leukemia through inter-laboratory sample distribution and comparison.

**Methods:**

This was a multi-center compatative trial study. A total of 37 samples with different BCR::ABL1^IS^ levels were uniformly prepared from patient bone marrow or peripheral blood to be discarded for comparison. Among them, 22 samples were used for real-time reverse transcription quantitative PCR（RQ-PCR）detection to revalidate the conversion factor（CF）, whereas another 15 samples, along with 6 samples used in RQ-PCR, were used for dPCR comparison. Peking University People's Hospital determined the validity of its own dPCR and RQ-PCR CFs by detecting World Health Organization international standards. Bland-Altman analysis was conducted to validate the CFs of participating laboratories. If no validated CF was available, the new CF was calculated using the current comparison samples, and its validity was assessed.

**Results:**

Among the 14 laboratories participating in the dPCR comparison, 13 detected BCR::ABL1 in all samples. Among the 13 laboratories participating in the RQ-PCR comparison, 5 had validated CFs, whereas the other 8 had validated CFs after recalculating. In the dPCR comparison, 11 laboratories used the same commercial kit, and 10 of them were qualified. Further, 1 laboratory used a laboratory-developed testing reagent and obtained a CF using the standard sample from the National Clinical Laboratory Center, which was also qualified, whereas the 2 laboratories with no CFs both had qualified CFs after calculating. Comparison of results from samples simultaneously performing dPCR and RQ-PCR demonstrated that the BCR::ABL1^IS^ results derived from their respective qualified CFs showed no significant difference for each laboratory（all *P*≥0.1）. However, among the 6 laboratories with unqualified original CFs in RQ-PCR, 2 showed significant differences between dPCR results and RQ-PCR results derived from the original CFs（*P*<0.05）, and 3 demonstrated tendencies toward significant differences（*P*>0.05 to <0.1）.

**Conclusion:**

dPCR detection of BCR::ABL1^IS^ shows high sensitivity and accuracy and has high consistency with RQ-PCR results.

酪氨酸激酶抑制剂（TKI）已成为慢性髓细胞性白血病（CML）慢性期患者的治疗基本药物。采用实时定量PCR（RQ-PCR）技术检测的BCR::ABL1（P210）mRNA水平代表了患者的分子学反应[1]–[2]，是疗效评估的基本指标。通过转换系数（CF）将各实验室的检测值转换为国际标准化BCR::ABL1 mRNA水平（BCR::ABL1^IS^），实现了分子学反应评估标准的全球统一[3]。北京大学人民医院、北京大学血液病研究所（简称北大人民）作为国内参比实验室，通过室间样本派发和比对在2011–2020年帮助全国七十余家实验室获得有效CF[4]–[6]。2025年，CF再验证即判定CF是否依然有效的工作在暂停5年后得以继续开展。

当前，停药实现无治疗缓解（TFR）已成为许多CML患者的治疗目标[1]–[2]。停药的前提是BCR::ABL1^IS^下降4.0或4.5个log，即获得分子学反应（MR）4.0或MR4.5[1]–[2]，由此对BCR::ABL1检测提出更高要求。最近几年数字PCR（dPCR）技术已走向成熟，其敏感性、特异性及准确性均较RQ-PCR有所提升。多项研究已证实dPCR相比RQ-PCR检测的BCR::ABL1^IS^能更好地预测停药成功[7]–[8]。由于国内dPCR的检测水平目前尚不可知，因此本次CF再验证工作首次加入了dPCR检测的室间比对。本文将展示dPCR检测的室间比对以及与RQ-PCR比较的结果。

## 材料与方法

1. 参与单位：本研究为多中心比对实验研究。共14家单位的实验室参加由北大人民发起的dPCR检测室间比对，其中13家同时参加基于RQ-PCR的CF再验证。本研究经北京大学人民医院伦理委员会批准（批件号：2022PHB103-001），豁免知情同意。

2. 样本制备、派发及检测：制备方法与以往CF确认及再验证相同[4]–[5]，即采用新鲜的临床送检RQ-PCR剩余待废弃的患者骨髓/外周血有核细胞制备。共制备出37种不同BCR::ABL1^IS^水平的比对样本，其中22种用于RQ-PCR的CF再验证，其余15种加上用于RQ-PCR的6种共21种样本用于dPCR检测比对。各家实验室各自进行RNA提取、逆转录及RQ-PCR和dPCR检测BCR::ABL1（P210）mRNA水平。

3. 北大人民CF的再验证：北大人民采用RQ-PCR和dPCR分别检测WHO用于定量BCR::ABL1（P210）的国际标准品（WHO授权英国国家生物标准与控制研究所生产，NIBSC 09/138），该标准品包含4份RNA样本，BCR::ABL1^IS^分别为0.01％、0.1％、1.0％及10.0％。结果分析与CF再验证流程相同。

4. RQ-PCR的CF再验证：回报RQ-PCR结果的实验室分别与北大人民之间进行BCR::ABL1^IS^的Bland-Altman一致性分析（GraphPad Prism 5.0）[4]–[5]，采用国际通用的标准判定合格，即偏倚在±1.2倍以内并且95％一致性范围在±5倍之间[9]。如果结果不合格或者因之前CF明显无法使用而未回报BCR::ABL1^IS^，则利用这批样本重新计算CF[10]，即将各实验室的检测值与北大人民BCR::ABL1^IS^之间建立回归方程：Logy＝（斜率×logMMR^IS^）+纵截距，计算得出CF后采用该CF转换为BCR::ABL1^IS^，然后再重复CF再验证的分析流程，如果达到标准，则该CF当前可用。

5. dPCR结果比对及结果分析：14家参与dPCR比对的实验室中12家回报了BCR::ABL1^IS^结果，它们与北大人民之间的比对采用了CF再验证的分析流程和判定标准。对于无CF的2家实验室，则采用计算CF的分析流程计算CF并评估有效性[10]。

6. dPCR与RQ-PCR结果的比较：采用配对*t*检验法对同时进行dPCR与RQ-PCR检测的6份样本进行结果的相关性分析。

## 结果

1. 北大人民CF的再验证：北大人民通过与澳大利亚医学和兽医科学研究所（IMVS）国际参比实验室样本比对获得有效CF而成为国内参比实验室。在本次比对中，北大人民首先检测了WHO国际标准品。RQ-PCR检测结果的偏倚为1.2倍，95％一致性范围为−0.9～1.3倍，因此CF依然合格，可继续用于本所临床检测中。对于本次比对，为了消除对其他实验室的CF评估引入偏倚而重新计算了CF，此CF对应的偏倚为1.0倍，95％一致性范围为−2.1～2.1倍，该CF转换的北大人民的BCR::ABL1^IS^结果用于本次CF再验证。

北大人民采用自带CF的商品化试剂盒进行dPCR（表1），WHO国际标准品检测的偏倚为1.0倍，95％一致性范围为−1.3～1.3倍，因此北大人民的BCR::ABL1^IS^用于本次dPCR比对。

**表1:** 参与比对的实验室数字PCR（dPCR）及实时定量PCR（RQ-PCR）比对结果

实验室编号	dPCR			RQ-PCR原有CF	dPCR与RQ-PCR结果比较（*P*值）	
	PCR试剂	CF来源	偏倚（95％一致性范围）（倍）		RQ-PCR合格或新计算的CF	RQ-PCR原有CF
北大人民	A试剂盒	试剂盒自带	1.0（−1.2～1.3）^a^	合格	0.463	–
L1	A试剂盒	试剂盒自带	−1.0（−2.0～1.9）	不合格	0.616	0.038
L2	A试剂盒	试剂盒自带	−1.0（−2.1～2.0）	不合格	0.285	0.088
L3	LDT	实验室自算	−1.0（−2.3～2.4）	合格	0.965	–
L4	A试剂盒	试剂盒自带	1.0（−1.9～2.0）	无	0.783	–
L5	A试剂盒	试剂盒自带	1.1（−1.8～1.9）	不合格	0.679	0.058
L6	A试剂盒	试剂盒自带	1.1（−2.0～2.1）	不合格	0.509	0.363
L7	A试剂盒	试剂盒自带	1.1（−1.9～2.0）	合格	0.106	–
L8	A试剂盒	试剂盒自带	1.1（−2.1～2.2）	不合格	0.240	0.071
L9	A试剂盒	试剂盒自带	−1.1（−2.4～2.2）	未做	–	–
L10	A试剂盒	试剂盒自带	1.1（−3.3～3.6）	不合格	0.403	0.021
L11	A试剂盒	试剂盒自带	1.1（−2.7～3.0）	无	0.366	–
L12	A试剂盒	试剂盒自带	−1.3（−2.6～2.0）	合格	–	–
L13	B试剂盒	无	−1.0（−2.5～2.4）^b^	合格	0.888	–
L14	LDT	无	1.2（−1.9～2.2）^b^	合格	0.107	–

**注** LDT：实验室自配试剂；CF：转换系数；–：①若RQ-PCR原有CF无则未与dPCR结果比较，②若RQ-PCR原有CF合格则与dPCR结果的比较放在左侧列；^a^来自与WHO标准品的比对；^b^来自重新计算CF的比对

2. 比对样本的BCR::ABL1^IS^组成：用于RQ-PCR比对的22份样本北大人民BCR::ABL1^IS^检测值分布如下：0.003 2％～0.01％（MR4.5～MR4.0）有3份、0.01％～0.1％（MR4.0～MR3.0）有10份、0.1％～10.0％（MR3.0～MR1.0）有9份，占比分别为14％、45％和41％。dPCR比对的21份样本BCR::ABL1^IS^分布如下：0.001％～0.003 2％（MR5.0～MR4.5）有8份，0.003 2％～0.01％（MR4.5～MR4.0）有7份、0.01％～0.1％（MR4.0～MR3.0）有6份，占比分别为38％、33％和29％，BCR::ABL1^IS^最低值为0.001 0％（MR5.0）。

3. 比对样本各家检测定性结果：各家实验室的RQ-PCR均能检测到BCR::ABL1，而dPCR结果中除了1家实验室（表1，L13）2份MR5.0～MR4.5的样本未检测到之外，其余均回报阳性结果。

4. RQ-PCR的CF再验证：参加RQ-PCR比对的13家实验室中9家采用两种商品化试剂盒、4家采用实验室自配试剂（LDT）。如表1所示，5家实验室CF合格（占38％）；6家CF不合格，2家未报告BCR::ABL1^IS^，这8家实验室重新计算的CF均达到合格标准。

5. dPCR结果比对：如表1所示，采用同一商品化试剂盒的11家实验室及采用LDT试剂并利用国家临床检验（临检）中心标准品获得CF的1家实验室回报了dPCR检测的BCR::ABL1^IS^结果，其中11家（占92％）结果合格，1家（L12）不合格；无CF的2家实验室（L13和L14）均可计算出合格的CF。

6. dPCR与RQ-PCR结果的比较：BCR::ABL1^IS^值介于MR4.5～MR3.0的6份样本同时进行了dPCR和RQ-PCR检测比对。如表1所示，13家实验室（除了未参加RQ-PCR的L9及dPCR不合格的L12）各自的dPCR与RQ-PCR合格或重新计算的合格CF得出的BCR::ABL1^IS^结果差异均无统计学意义（均*P*≥0.1；以L1和L10为例见图1A和1B）；然而RQ-PCR原有CF不合格的6家实验室中，2家的dPCR与由原有CF得出的BCR::ABL1^IS^结果之间差异有统计学意义（L1和L10，均*P*<0.05，图1C和1D），3家趋于差异有统计学意义（L2、L5和L8，0.05<*P*<0.1）。

**图1:**
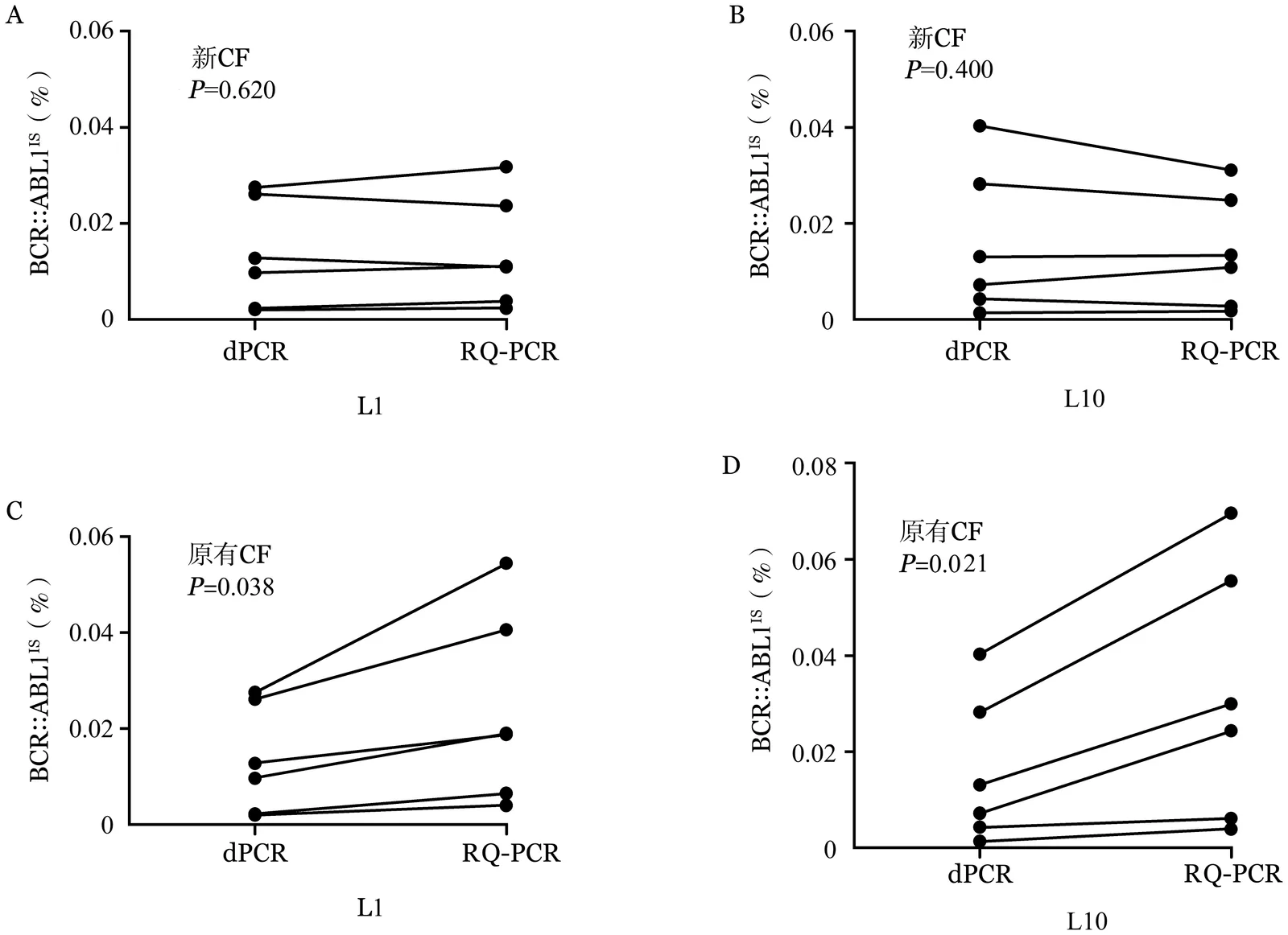
同一实验室6份样本数字PCR（dPCR）与实时定量PCR（RQ-PCR）结果的比较 **A** 实验室L1，采用重新计算CF得出的RQ-PCR结果；**B** 实验室L10，采用重新计算CF得出的RQ-PCR结果；**C** 实验室L1，采用原有CF得出的RQ-PCR结果；**D** 实验室L10，采用原有CF得出的RQ-PCR结果

7. dPCR检测的BCR::ABL1^IS^的室间差异：分别计算dPCR比对的21份样本各家BCR::ABL1^IS^结果的平均值及变异系数（CV），如图2所示，二者呈明显负相关（Spearman相关系数*r*＝−0.48，*P*＝0.027）。

**图2:**
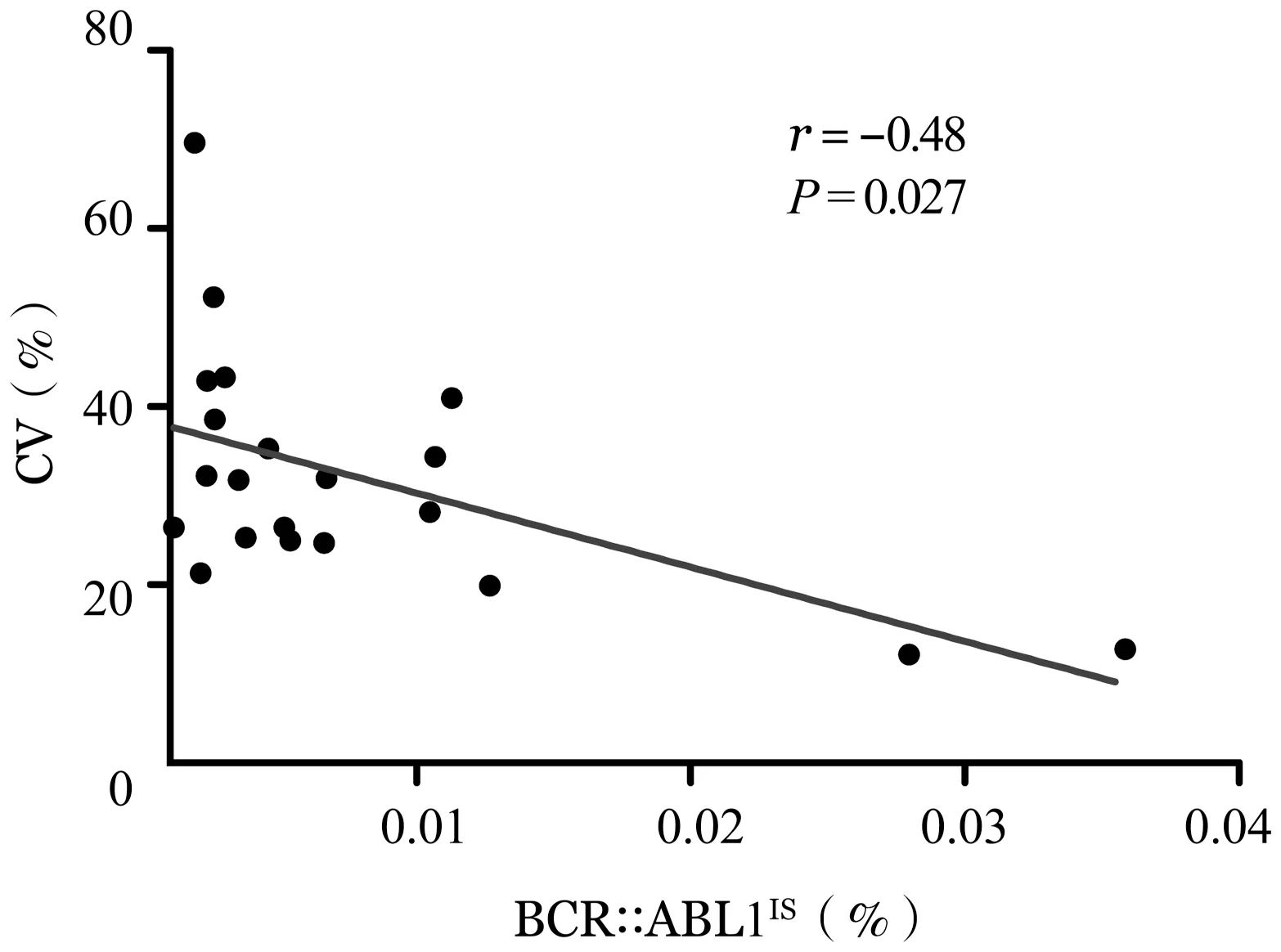
数字PCR（dPCR）比对的21份样本各实验室BCR::ABL1^IS^结果的平均值与其变异系数（CV）之间的关系

## 讨论

RQ-PCR仍然是当前广泛使用的监测CML患者BCR::ABL1 mRNA水平的常规技术，但其无法精准评估患者是否达到停药要求的分子学反应。欧洲白血病网（ELN）于2025年发表的CML管理推荐认为，dPCR由于其技术优势是一种可以接受的替代方法，尤其是在很低水平的疾病状态时[1]。因此，有必要对国内实验室dPCR的检测能力予以评估。室间样本派发和比对既是分子检测室间质评的基本手段，也是获得用于转换BCR::ABL1^IS^的CF的经典方式[9]，[11]。CF获得后需要定期进行再验证以保证其持续有效。2025年度我们重启了基于RQ-PCR的CF再验证并首次开展dPCR比对。这两种比对均采用了与以往CF再验证相似的流程，即采用患者样本制备20余份比对样本，各参与实验室将比对样本与常规临检样本同时检测并分散进行。这样是为了接近临检场景、反映临检水平和能力，以保证获得的CF真正适用于临检CML患者的BCR::ABL1^IS^转换。对于CF再验证不合格的实验室，我们利用这些样本的结果重新计算CF并评估其转换的BCR::ABL1^IS^，如果合格则该CF为该实验室当前有效CF，以实现当前临检准确报告BCR::ABL1^IS^。

北大人民采用拥有注册证的商品化dPCR试剂盒检测WHO国际标准品的偏倚为1.0倍，说明几乎无偏倚，从而证实其检测BCR::ABL1^IS^的高准确性。dPCR比对全部为MR3.0之下即深层分子学反应的样本，其中2/3为MR4.0以下，最低为MR5.0。而BCR::ABL1水平越低则变异越大[12]，本次比对结果也证明了这一点（图2）。参与dPCR比对的实验室中12家采用北大人民同样的试剂盒，均能检测到全部样本BCR::ABL1，并且比对结果仅1家不合格。以上结果说明，试剂是保证检测准确的主要因素，在采用可靠试剂的前提下，dPCR能够稳定检测至MR5.0并且不同实验室的结果可达到高度的一致性。1家通过LDT方式实施dPCR并通过其他渠道获得CF的实验室的dPCR比对也合格（表1，L3）。这样不同来源的结果互相印证了各自dPCR检测BCR::ABL1^IS^的准确性，也证明了本次比对的可靠性。

6份样本同时用于dPCR与RQ-PCR比对。结果显示，dPCR合格并参与RQ-PCR比对的13家实验室（包括北大人民）各自dPCR与用合格CF转换的RQ-PCR结果之间差异均无统计学意义，相比之下，6家用RQ-PCR不合格的CF转换的BCR::ABL1^IS^中5家与dPCR之间存在或趋于存在显著性差异，说明只要是合格的CF转换的BCR::ABL1^IS^，dPCR与RQ-PCR之间结果就是一致的。因此，在合格CF的前提下，CML患者RQ-PCR与dPCR检测的BCR::ABL1^IS^结果之间具有可比性，可无缝连接评估其分子学反应变化趋势。

本次国内实验室间BCR::ABL1^IS^的dPCR比对显示了dPCR检测的高敏感性、高准确性，以及与RQ-PCR之间的高度一致性，为其临床应用提供了依据。dPCR检测的高敏感性为其性能验证提出更高要求，因此采用商品化试剂盒是主流，国内现状亦如此。目前已有国产的dPCR仪及dPCR检测试剂盒获得国家药监局注册证，这为该检测的临床广泛应用奠定基础。此外，由于成本高导致的临床收费高等因素的限制，目前尚建议dPCR用于获得深层次分子学反应后并有停药需求的CML患者临床分子监测。
